# An Engineering Approach to Biomedical Sciences: Advanced Strategies in Drug Delivery Systems Production

**Published:** 2012-10-11

**Authors:** Anna Angela Barba, Annalisa Dalmoro, Matteo d’Amore

**Affiliations:** 1 Dipartimento di Scienze Farmaceutiche e Biomediche, Università di Salerno, Salerno, Italy; 2 Dipartimento di Ingegneria Industriale, Università di Salerno, Salerno, Italy aabarba@unisa.it, www.minerva.unisa.it

**Keywords:** process intensification, ultrasonic atomization, microwave heating

## Abstract

Development and optimization of novel production techniques for drug delivery systems are fundamental steps in the “from the bench to the bedside” process which is the base of translational medicine. In particular, in the current scenery where the need for reducing energy consumption, emissions, wastes and risks drives the development of sustainable processes, new pharmaceutical manufacturing does not constitute an exception. In this paper, concepts of process intensification are presented and their transposition in drug delivery systems production is discussed. Moreover, some examples on intensified techniques, for drug microencapsulation and granules drying, are reported.

## I. INTRODUCTION

The final step of the translational medicine chain is the production of medical/pharmaceutical devices efficient and widespread for *in health maintenance* of patients. Under the production point of view, current approaches in the manufacturing of drug delivery systems are focused on the dual goal:

– to optimize manufacturing processes in response to the need for reducing energy consumption, emissions, wastes and risks;
– to innovate the functionality of new formulations using biocompatible materials.

Within this frame, several researches were carried out in our group (*Transport Phenomena and Processes, www.minerva.unisa.it*) with the aim of bringing the engineering point of view on innovation in the development of pharmaceutical techniques. Key-word of this innovation is “process intensification”: meaning and applications are reported in the following.

## II. PROCESS INTENSIFICATION

**Fig 1:**
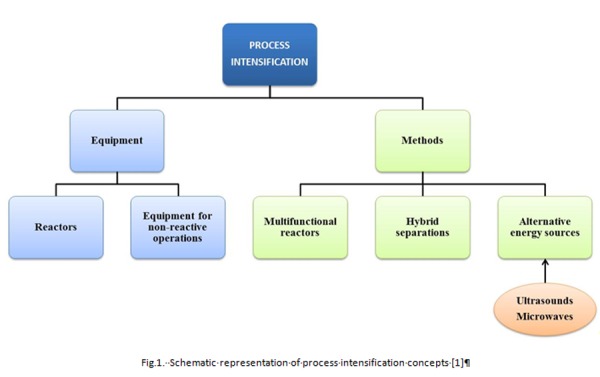
Schematic representation of process intensification concepts [1]

The current industrial scenery is founded on compromises based on the needs of the industrial processes developed to satisfy both the increasing market requirements and the mandatory rules in sustainable productions such as raw material / energy savings, respect of environmental constraints of industrial-scale processes [1]. In this frame, process intensification represents an engineering tool in looking for safer operating conditions, lower wastes and costs, higher productivity and developing of multifunctional devices [2, 3]. At last this new manufacturing vision was defined as an advanced *strategy which aims to achieve process miniaturization, reduction in capital cost, improved inherent safety and energy efficiency, and often improved product quality*[4]. Moreover, benefits of process intensification include simpler scale-up procedures, and possibility to allow the replacement of batch processing by small continuous reactors, which frequently give more efficient overall operation [5]. Finally, process intensification contains not only the development of novel, miniaturized equipments but also intensified methods of processing which may involve the use of ultrasonic (US) and radiation energy sources such as radio frequencies (RF) and microwaves (MW) [4]. Process intensification toolbox is reported in Fig. 1.

In this work ultrasonic atomization technique and heating treatment assisted by microwave, applied in developing of drug delivery systems, are the matter of discussion. Both the processes constitute tools for process intensification since they involve much less energy with respect to conventional techniques commonly adopted in atomization of liquid and heating purposes, respectively. Moreover, due to their peculiar features that will be elucidate in the following sub-sections, uses of US and MW can address towards miniaturization of process chambers, reducing maintenance costs, and minimize denaturation risks due to thermal/mechanical stress of sensible materials such as pharmaceuticals [1], [6], [7].

## III. METHODOLOGY

### A. Microencapsulation by ultrasonic atomization

Microencapsulation of drugs is one of the manufacturing techniques that guarantees the overcoming of serious problems associated to the delivery of drugs with poor stability in physiological environments and scarcely absorbable. Relevance of encapsulation techniques consists in the effectiveness to protect different kinds of active molecules (drugs, gene, vitamins and proteins) through their envelopment in suitable carriers [1]. Microencapsulation can be performed by both physical-chemical and mechanical processes [8–10] all based on these fundamental steps: incorporation of active molecules in opportune bio-compatible carriers; droplets formation; solvent removal; microparticles harvest and stabilizing treatment [11]. Among the mechanical processes, micro-encapsulation by ultrasonic assisted atomization (fine droplets formation) is characterized by several interesting features [12]. Briefly, ultrasonic waves (ultrasonic frequency allowed: 18–100 kHz) are mechanical phenomena that can induce stress in material medium by vibrational energy. By ultrasonic atomizer very small droplets with uniform size may be formed by feeding a fluid, at a controlled rate, through a small orifice in the tip of a horn vibrating ultrasonically. Vibration energy causes the breakup of liquid in threads and then of droplets. In particular, phenomena of cavitation and capillary-wave are the two rival hypothesized mechanisms to explain fine droplets generation [13].

It is important to note that final dimensions of fine droplets are affected from the frequency of the adopted ultrasonic waves: an increase in frequency produces more fine particles.

**Fig. 2:**
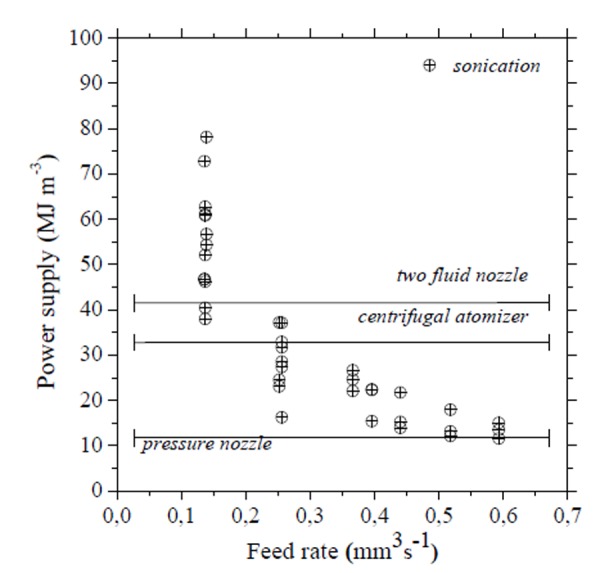
Energy request for different atomization devices (bars are referred to the exercise of conventional atomizers). Working at high flow rates (typical condition in plant scale process) ultrasonic assisted atomization is confirmed to be a very promising technique in the intensification of industrial processes which includes an atomization step [13].

The relevance of the ultrasonic atomization can be mainly attributed to its ability to produce drops of small size and low inertia since it works involving low velocities. This feature allows reducing the energy request respect to the more conventional atomization systems (nozzle atomizers, two fluid atomizers) (Fig. 2), the volume of apparatuses (compact equipment) and the mechanical stress of treated feeds avoiding deactivation of sensitive substances such as pharmaceuticals [1, 13].

The role of several operating parameters (solution concentration, flow rate, atomization power) in atomization process and correlations able to predict the features of the droplets as functions of process parameters were investigated and reported in [13].

Starting from aqueous solutions of biocompatible polymers, used as drug carriers, microencapsulation assisted by ultrasonic atomization was applied to develop delivery systems loaded by hydrophilic or lipophilic model molecules. In particular, solutions of chitosan or alginate loaded respectively with B12 vitamin (hydrophilic model molecule) and α-tocopherol vitamin (TOC, lipophilic model molecule) were used to produce microparticles by two different ultrasonic processors (*VCX 130 PB 130 W, 20 kHz, Sonics & Materials Inc., CT, USA; broadband ultrasonic generator mod. 06-05108-25 KHz Sono-Tek corporation, NY, USA*) and tips (*sonotrode VC 4020 50 W, 20 kHz, Sonics & Materials Inc., CT, USA* single channel; dual liquid feed probe *Sono-Tek 025-00010, Sono-Tek corporation NY, USA*) as sources of ultrasonic vibration and atomizer, respectively.

**Fig. 3:**
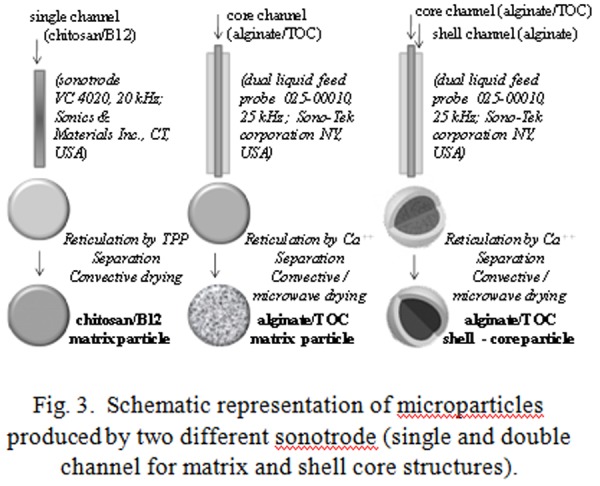
Schematic representation of microparticles produced by two different sonotrode (single and double channel for matrix and shell core structures).

Chitosan/B12 feed solution was delivered to the single channel sonotrode by a peristaltic pump (*Velp Scientifica Italia, Usmate, Milan, IT*). The produced fine drops were collected in the reticulating bulk (sodium tripolyphosphate solution, TPP) placed on a magnetic stirrer. By crosslinking phenomena between chitosan polymeric chains and P_3_O_10_^5−^ ionic groups of tripolyphosphate fine rubbery particles were obtained (matrix structures of chitosan/B12) (Fig. 3).

Alginate/TOC (in core channel) and alginate/TOC (in core channel) / alginate (in shell channel) feed solutions (Fig. 3) were delivered to the double channel sonotrode by peristaltic pumps (*Verderflex OEM mod. Au EZ, RS Components Milan IT*, controlled by two systems with variable tension *Long WEI DC power supply PS305D* to adjust the delivered solutions flow rate). The produced fine drops were collected in the reticulating bulk (calcium chloride solution) placed on a magnetic stirrer. By crosslinking phenomena between alginate polymeric chains and bivalent calcium cations, fine particles with matrix and shell - core structures are obtained (Fig. 3).

All kinds of produced microparticles were separated by a filtration process, washed, photographed (by Canon digital camera, IXUS 850 IS, by Leica digital camera DFC 280 mounted on optical microscope Leica Microsystems DM-LP, Wetzlar, D) and subjected to diameter measurements by image analysis (using the public domain software ImageJ 1.40g, Wayne Rasband, National Institutes of Health, USA, freely available at http://rsb.info.nih.gov/ij/). Finally, produced particles were stabilized by convective drying in oven (ISCO 9000) at 45°C or by microwave drying (commercial microwave oven De Longhi, mod. Easy max power 700W) until reaching constant weight. In Fig. 3 the fundamental steps of production were shown.

At last, stabilized loaded microparticles were subjected to dissolution tests to evaluate the yield of encapsulation, and the release kinetics under physiological conditions (USP II apparatus, 37°C; 100 rpm paddle rotating speed; for chitosan particles: buffer solution at pH 7.4; for enteric alginate particles: two stages protocol in buffer solutions at pH 1 and pH 6.8). All the tests were performed in triplicate.

### B. Microwave assisted drying

Interest in applications of microwave power in pharmaceuticals treatments (i.e. heating, drying, curing) is relatively recent and not well widespread [7]. A non-consolidated know-how in microwave application for pharmaceutical products and, under a more technical point of view, the need to have a uniform distribution of heat to avoid local overheating of the material, and, moreover, the possibility of a temperature uncontrolled increase are the main limiting factors [6]. In the last twenty years, mainly in Academic researches and in a few large-scale plants experiences, microwaves benefits have begun to capture attention. In particular, microwaves have been:

– applied in modulating drug and excipient properties via specific material microwave interactions [15, 16];
– tested in drying operations of powder and granules [17, 18];
– used in single-pot devices to carry out mixing, granulation and drying of pharmaceuticals in one vessel [17, 19, 20].

Microwaves are electromagnetic radiation with frequency ranging from 300 GHz to 300 MHz; uses of the different parts of the spectrum are regulated by international agreement. In particular, the frequencies 0.915 and 2.45 GHz are the most common frequencies dedicated to microwave power applications for industrial, scientific and medical purposes [21]. Reasons for the growing interest of microwave heating can be found in the peculiar mechanism for energy transfer: during microwave heating, energy is delivered directly to materials through molecular interactions with electromagnetic field via conversion of electrical field energy into thermal energy. This can allow unique benefits, such as high efficiency of energy conversion and shorter processing times, thus reductions in manufacturing costs thanks to energy saving. Moreover, other effects have been pointed out, such as the possibility to induce new structural properties to irradiated materials (development of new materials) and to apply novel strategies in chemical syntheses (green techniques) [7].

Crucial parameters in microwave heating are the dielectric properties of matter; they express the energy coupling of a material with electromagnetic microwave field and, thus, the heating feasibility [7, 21]. In particular, the ability of a material to interact with electromagnetic energy is related to the material’s complex permittivity (dielectric properties or susceptibility). This property is characterized by a frequency-depending and is reported as a relative complex number: ε_r_ =ε/ε_0_ = ε’-j ε” where ε_0_ is the vacuum permittivity (ε_0_ = 8.85 10^−12^ F/m); ε’ is the part named dielectric constant and ε” is the imaginary part known as loss factor. The dielectric constant is a measure of how much energy from an external electric field is stored in the material; the loss factor accounts for the loss energy dissipative mechanisms in the material. Therefore, a material with a high loss factor is easily heated by microwave.

**Fig. 4:**
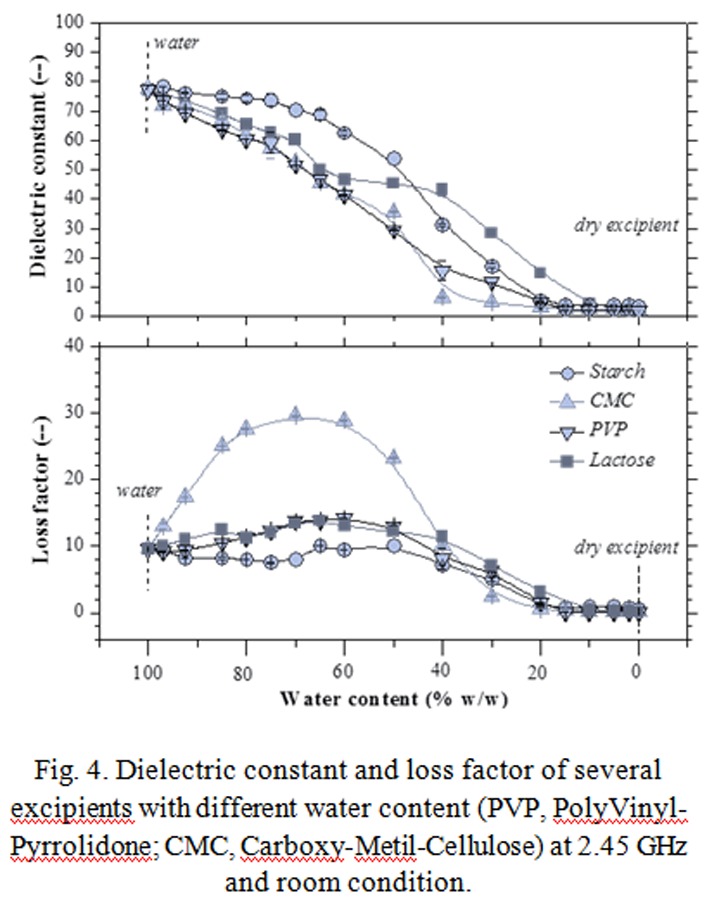
Dielectric constant and loss factor of several excipients with different water content (PVP, PolyVinyl-Pyrrolidone; CMC, Carboxy-Metil-Cellulose) at 2.45 GHz and room condition.

Wet / liquid pharmaceutical dosage forms (hydrogel, ointments, syrups) constitute dissipative materials; generally, powder pharmaceutical ingredients are poor dissipative constituents but when solvents (water, alcohols, hydro-alcohol mixtures) are involved in their processing, the mixtures permittivity generally increases and thus this allows efficacious microwave heating. In Fig. 4 dielectric properties at 2.45 GHz of different mixtures of water / powder pharmaceutical excipients are reported. As one can see, nature of excipients and water content determine the microwave heating capability. Thus, on the bases of dielectric properties knowledge, optimized working protocols must be applied.

Powders of hydroxypropyl methyl cellulose (HPMC, a biocompatible polymer used in formulating of hydrogel based dosage forms) were used to produce a model granular pharmaceutical dosage system dried by microwave heating. Water / ethanol mixture (50% v/v) was used as wet binder phase in wet granulation process carried out in a bench-scale mixer (*Mini Mixer MULTI LAB Caleva Process Solutions Ltd, UK*). Granular product was then characterized in terms of size (by image analysis, binder phase content (*Ohaus moisture balance mod MB45*), dielectric properties (*network analyzer Agilent Tecnologies mod ES 8753 and coaxial probe Agilent Tecnologies mod 85070D*). By a multimodal microwave cavity (*LBP 210/50 Microwave Oven 2300 W, InLand, USA*) different drying treatments were performed to simulate stabilization processes (by reducing the binder phase content) allowed in industrial manufacturing to guarantee firstly granules inalterability and flowability.

Drying kinetics was studied to emphasize feasibility and advantages of microwave assisted drying in terms of reduces process time and thermal stresses in comparison to classic convective heating treatments (convective granules drying in the conventional oven *ISCO 9000;* temperature measurements were carried out by infrared pyrometer *Simpson mod. IR-10*). All the tests were performed in triplicate.

## IV. RESULTS AND DISCUSSION

### A. Microencapsulation results

**Fig. 5:**
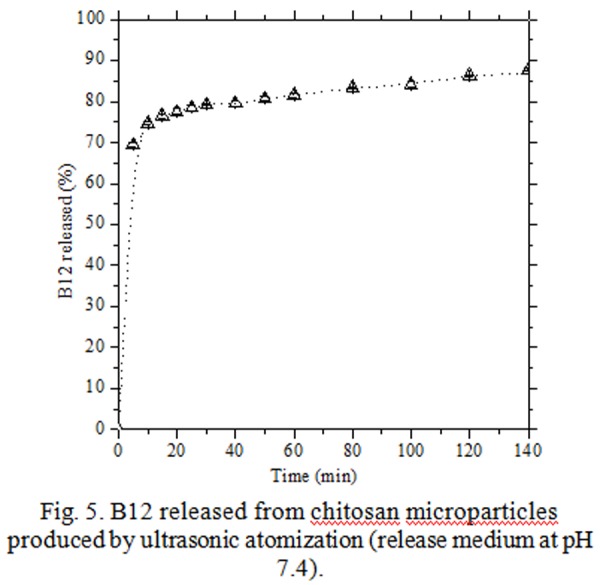
B12 released from chitosan microparticles produced by ultrasonic atomization (release medium at pH 7.4).

Chitosan/B12 microparticles produced by the single channel sonotrode (20 kHz), after convective treatments (air bulk temperature: 45°C) showed a spherical morphology and an average size of 600 μm. The yield of encapsulation (amount of B12 released and measured / theoretical loaded amount of B12) was found around 12 %. In Fig. 5 the B12 release profile was reported.

The release kinetics showed a rapid burst of B12 in the aqueous bulk (pH 7.4): in the first 5 min, the released B12 was already over 70%; a further increase of 10% was monitored in the successive half hour; after 140 min a percentage of release of around 90% was achieved.

These results were attributed mainly to the network large *mesh-size* obtained in reticulation step between TPP ionic groups and chitosan chains that allows the fast diffusion of B12 from the dried particles to the dissolution bulk [22].

Alginate particles, both matrix (only core, without protective shell) and shell-core, produced by double channel sonotrode (25 kHz), showed a spherical morphology an average size of 70 μm. The yield of encapsulation (amount of TOC released and measured / theoretical loaded amount of TOC) was found around 100 %.

**Fig. 6:**
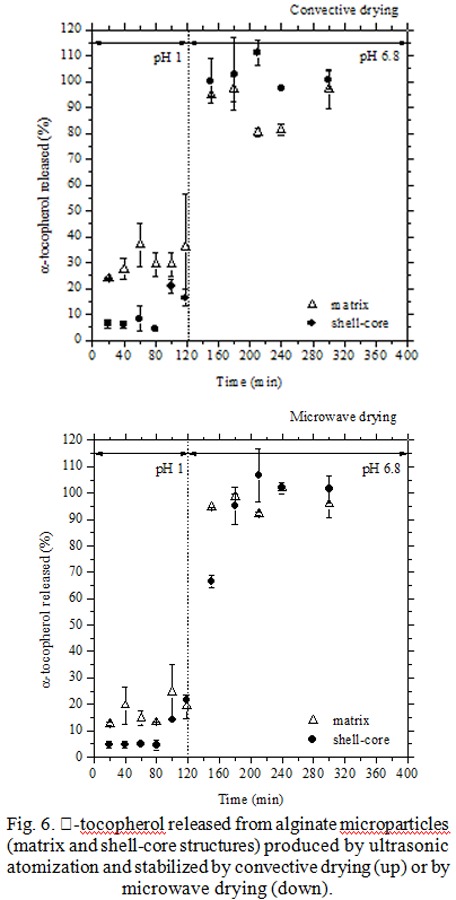
α-tocopherol released from alginate microparticles (matrix and shell-core structures) produced by ultrasonic atomization and stabilized by convective drying (up) or by microwave drying (down).

The two kinds of microparticles were stabilized adopting both convective (conventional treatment at 45°C overnight) and microwave drying (novel treatment, at pulsed irradiation in about 30 min) with the aim to observe the kinetic rates and possible changes in the release properties. The release profiles of both matrix and shell-core microparticles (Fig. 6) globally showed that by microwave treatments a little delay in TOC release was achieved. This demonstrates the microwave drying usefulness in the controlled drug release: it can affect, by a sort of curing, the polymeric structures.

The enteric test (pH 1 for two hours, to simulate gastric conditions, then pH 6.8, i.e. intestine pH) on both kinds of microparticles showed different gastroresistance properties. In particular, shell-core microparticles have assured a better protection of active principle at initial stage of pH 1, releasing only the 5% of α-tocopherol against the 10–20% for matrix particles. Moreover, matrix particles have released all the loaded α-tocopherol as soon as the pH changes from pH 1 to pH 6.8. On the contrary, shell-core microparticles were characterized by a delayed release.

Summarizing, the combination of two powerful tools of process intensification, ultrasonic atomization and microwave drying, gives to produced microsystems peculiarities (high loading, delayed release) that make them of interest for specific drug delivery applications.

### B. Microwave assisted drying results

Model granules of HPMC/hydro-alcoholic binder with a wet phase (or briefly moisture) content around 31% (wb, wet bases) were subjected to both convective and microwave drying treatments. To this aim given amounts of granules were placed in a Petri dish and undergone to weight and temperature measurements after different process times. To compare all the drying curves (moisture *vs* time) residual moisture was expressed as moisture ratio, *MR*:

(1) $$\mathrm{MR}(t)=\frac{M(t)-M_{e}}{M_{0}-M_{e}}$$

where *M(t)*, *M_e_* and *M_0_* are the actual, the equilibrium and the initial moisture, respectively.

**Fig. 7:**
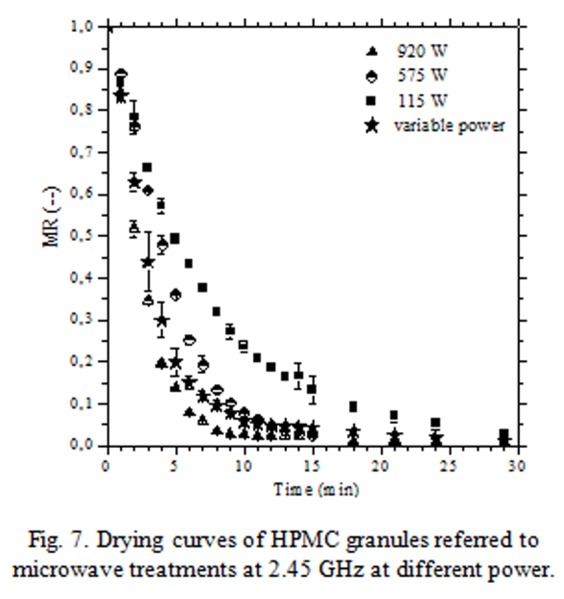
Drying curves of HPMC granules referred to microwave treatments at 2.45 GHz at different power.

**Fig. 8:**
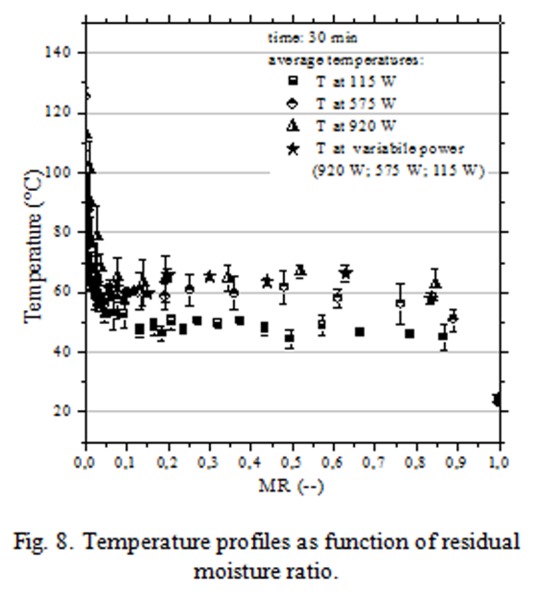
Temperature profiles as function of residual moisture ratio.

In Fig. 7 were reported the drying curves obtained by microwave assisted heating at different power (115 W; 575 W and 920 W). It is possible to note that the stabilization process (completed when the granules wet phase was reduced at 2–3% wb) occurred in 15–30 min depending on the applied power intensity (920 W - 115 W). “Variable power” label referred to a controlled power protocol developed to irradiate, at maximum power intensity, granules with high moisture content and at lowest power intensity, almost dried granules. By this way granules temperature was kept at low values avoiding thermal stresses; sudden increases were observed only at the end of drying treatments (Fig. 8).

It is important to note that microwave apparatus was not interested by heating phenomena because heat was directly generated in wet HPMC granules by dissipative mechanisms due to the dielectric properties of the hydro-alcoholic binder phase (dielectric properties: wet granules ε = 3,24 - j 1,07; dried granules ε =1,90 - j 0,17).

**Fig. 9:**
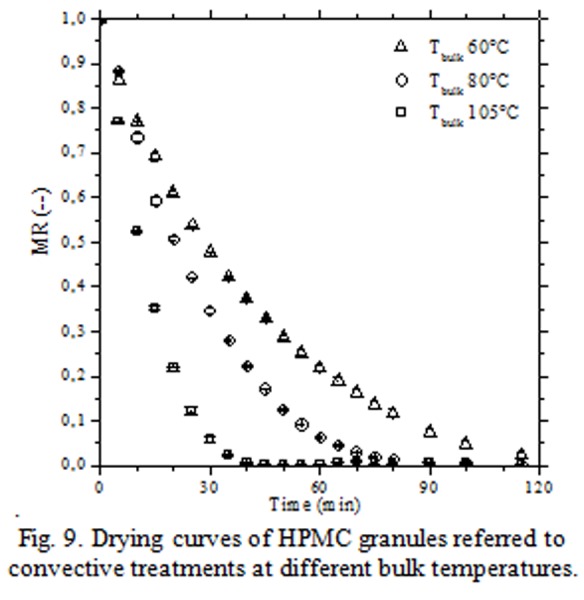
Drying curves of HPMC granules referred to convective treatments at different bulk temperatures.

**Fig. 10:**
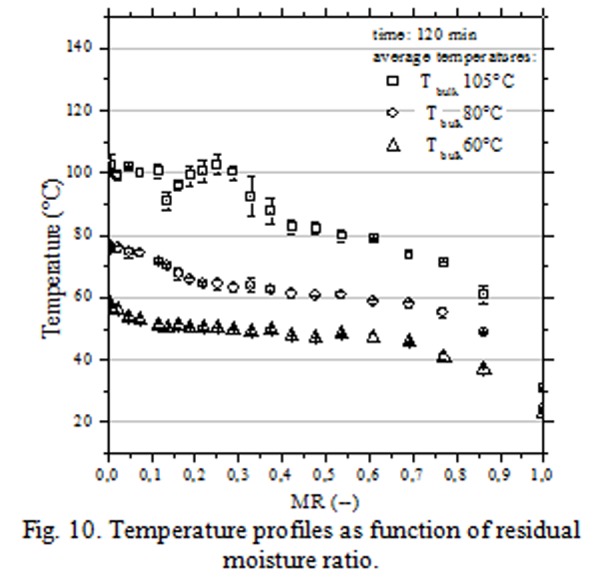
Temperature profiles as function of residual moisture ratio.

In Fig. 9 were reported the drying curves obtained by convective treatments at different temperature of air bulk (60°C; 90°C and 105°C). Stabilization process occurred in 30 – 120 min depending on the air bulk temperature (105 – 60°C); globally, granules were exposed to high temperature for prolonged time (Fig. 10).

Dried model granules, treated both via convective methods and through microwave processes, showed a roughly spherical morphology and an average size of 1,55 mm, features very close to the values of the wet granules. Moreover their final flowability was found good (dried granules Carr’s index ≅ 17%; HPMC powders Carr’s index ≅ 33%, where a Carr’s index until 20% denotes good material flowability).

Summarizing, the relevance of the microwave treatments was highlighted by the shorter process time and by e availability of an *ad hoc* process parameter (power intensity) to be tuned to minimize thermal damage.

## V. CONCLUSIONS

Translational medicine is based on a multidisciplinary approach for the conversion of basic science findings into novel therapies. Indeed, the engineering point of view could be useful in defining novel preparation techniques for drug delivery systems.

In this work, two different applications of ultrasonic and microwave energy were presented (part A and part B).

In the part A, the production and the characterization of three kinds of microparticles produced by ultrasonic atomization were reported. Both chitosan and alginate polymer solutions were successfully atomized producing microparticles loaded with different active molecules.

In the part B, comparison between the drying kinetics of wet HPMC granules carried out by convective and microwave treatments was performed highlighting that microwave heating requires shorter process time and on the bases of material dielectric properties, gives the availability of an *ad hoc* process parameter (power intensity) to be tuned to minimize possible thermal damage.

Both the applications are of great technological interest, then our research is currently focused on these issues.
